# Perceived and Objective Measures of Neighborhood Environment for Physical Activity Among Mexican Adults, 2011

**DOI:** 10.5888/pcd13.160009

**Published:** 2016-06-09

**Authors:** Alejandra Jáuregui, Deborah Salvo, Héctor Lamadrid-Figueroa, Bernardo Hernández, Juan A. Rivera-Dommarco, Michael Pratt

**Affiliations:** Author Affiliations: Deborah Salvo, Centro de Investigación en Nutrición y Salud, Instituto Nacional de Salud Pública, Cuernavaca, Morelos, México, School of Public Health, University of Texas, Austin, Texas; Héctor Lamadrid-Figueroa, Centro de Investigación en Salud Poblacional, Instituto Nacional de Salud Pública, Cuernavaca, Morelos, México; Bernardo Hernández, Institute for Health Metrics and Evaluation, University of Washington, Seattle, Washington; Juan A. Rivera-Dommarco, Centro de Investigación en Nutrición y Salud, Instituto Nacional de Salud Pública, Cuernavaca, Morelos, México; Michael Pratt, Rollins School of Public Health, Emory University, Atlanta, Georgia.

## Abstract

**Introduction:**

Environmental supports for physical activity may help residents to be physically active. However, such supports might not help if residents’ perceptions of the built environment do not correspond with objective measures. We assessed the associations between objective and perceived measures of the built environment among adults in Cuernavaca, Mexico, and examined whether certain variables modified this relationship.

**Methods:**

We conducted a population-based (n = 645) study in 2011 that used objective (based on geographic information systems) and perceived (by questionnaire) measures of the following features of the built environment: residential density, mixed-land use, intersection density, and proximity to parks and transit stops. We used linear regression to assess the adjusted associations between these measures and to identify variables modifying these relationships.

**Results:**

Adjusted associations were significant for all features (*P* < .05) except intersection density and proximity to transit stops. Significantly stronger associations between perceived and objective measures were observed among participants with low socioeconomic status, participants who did not own a motor vehicle or did not meet physical activity recommendations, and participants perceiving parks as safe.

**Conclusion:**

Perceived measures of residential density, mixed-land use, and proximity to parks are associated with objective environmental measures related to physical activity. However, in Mexico, it should not be assumed that perceived measures of intersection density and proximity to transit stops are the same as objective measures. Our results are consistent with those from high-income countries in that associations between perceived and objective measures are modified by individual sociodemographic and psychosocial factors.

## Introduction

Urban design and re-engineering of infrastructure are important potential strategies for promoting physical activity (PA) (1). Providing safe, attractive, and convenient settings for PA may help residents incorporate PA into their lives and meet PA recommendations (2). However, improving features of the built environment may not be sufficient to motivate residents. The decision to engage in PA may result from direct or indirect influences of the built environment and may be mediated by individual cognitive factors (3), such as people’s perceptions about their environment (4).

Individuals’ perceptions of the environment are measured via self-report, whereas objective measures are generally derived from data produced by geographic information systems (GIS) or street audits. Perceptions are filtered through individual standards of evaluation (5); thus, 2 individuals may perceive the same environment differently.

Studies examining the correspondence between perceived and objective measures of the built environment in high-income countries show poor to moderate agreement (κ = 0.01–0.49), and results vary by feature and setting (4,6–10). The effect of the built environment on PA may depend on the level of agreement between perceived and objective measures of the environment (11). Low correlation between objective and perceived measures of the built environment has been found among older adults (4), people who have low socioeconomic status (SES) (6), married or cohabitating adults (4), people who have children in the household (6), and those who engage in low levels of PA (10). Walking distance to the nearest supermarket is overestimated to a greater extent by men than by women (12).

In low- and middle-income countries, evidence on the correspondence between objective and perceived measures of the built environment is scarce. The aim of this study was to test the correspondence between objective and perceived measures of the built environment for PA in a representative sample of adults from Cuernavaca, Mexico, and to assess whether certain variables modify these relationships.

## Methods

We conducted a cross-sectional, population-based study of adults in Cuernavaca, Mexico (population, 365,000) in 2011. A representative sample of Cuernavaca residents was selected by using census tracts as primary sampling units. Cuernavaca is divided into 123 census tracts, which were stratified into 4 levels of SES determined by the National Institute of Statistics, Geography and Informatics (INEGI) (13) and 2 levels of objectively measured walkability (14). Eight census tracts per stratum were randomly selected, yielding 32 study census tracts. Seven blocks were randomly selected per census tract and 2 to 4 households were selected per block (15).

Field workers recruited one participant per household during a home visit. Eligible participants were aged from 20 to 65 years, able to walk, and permanent residents of that household. Details on sampling strategy and data collection are available elsewhere (15). The study response rate was 58.9% (677/1,150; based on the number of selected households with an eligible adult). Of the 677 participants originally recruited, 18 had incomplete data on environmental perceptions, 6 had incomplete data on objectively measured features, and 8 did not meet accelerometry criteria, leaving 645 for analysis. The study was approved by the institutional review boards of Emory University and the Mexican National Institute of Public Health.

We used the Abbreviated Neighborhood Environment Walkability Scale (ANEWS) adapted for use in Latin America for measuring perceptions of environmental features (16). ANEWS measures perceptions of environmental features hypothesized to be related to PA, including land-use mix, intersection density, residential density, proximity to transit stops, proximity to parks, perceived neighborhood safety, and perceived park safety (as well as others that were not used in this analysis). “Land-use mix” refers to the diversity of destinations (eg, grocery stores, post offices, parks) within walking distance of a person’s residence. “Intersection density” refers to street connectivity: as density increases, more walking routes are available (with implications for increased safety) and walking for transportation becomes more interesting and efficient. “Residential density” refers to the critical mass of people: an increase in residential density increases the number of people who can be active and the opportunities for people to see others being active. Walking to and from transit stops offers an opportunity to be active. Studies demonstrate good test–retest reliability for ANEWS (intraclass correlation coefficient > 0.75) (10,17).

For objective measures of environmental features, the location of each participant was manually geocoded in ArcGIS (ESRI, Inc). We defined 500-m and 1-km street-network buffers around each participant’s residence. Similar buffers were reported to adequately capture data on perceptions among adults of neighborhood walkability (9,18,19). Data sources were provided by INEGI and the Land Use Registry Department of the City of Cuernavaca (20).

For each feature, we computed an objective variable consistent with the feature measured by ANEWS (Appendix). For example, for residential density, ANEWS asks participants about 6 types of residential buildings and then generates a residential density score based on the number of single family units per buffer area; our objective measure of residential density was a count of residential units instead of a residential density score.

Self-reported data were collected on age, sex, education level, marital status, individual SES (based on 25 questions on household features and assets used by the National Health and Nutrition Surveys of Mexico [23]), and motor vehicle ownership. Minutes per week of moderate to vigorous PA were measured with GT3X Actigraph accelerometers (ActiGraph, LLC) using 60-second epochs and scored by using the cut points for adults defined by Freedson et al (24). We obtained summary scores for perceived neighborhood safety and perceived park safety using ANEWS; these variables were dichotomized as safe or unsafe. Details are available elsewhere on how data on these variables were collected and processed (15,25).

### Statistical analysis

Descriptive statistics (means, 95% confidence intervals [CIs], ranges, and percentages) were estimated for each perceived and objective variable. To test the correspondence between objective and perceived measures, we estimated Pearson correlations between continuous variables using the *corr_svy* procedure (26). Because we observed positively skewed distributions in perceived and objective measures of residential density and proximity to transit stops, we log-transformed these variables before calculating correlations.

We used multivariate regression models to estimate associations between objective and perceived measures of the built environment. Perceived residential density and proximity to transit stops were treated as natural-log-transformed variables because this parameterization provided the best fit. First, we ran unadjusted linear regression models with the perceived measure as the dependent variable and the objective measure as independent variable. Exploratory analyses suggested nonlinear relationships between all objective and perceived measures of the built environment; therefore all objective variables were introduced as quintiles (using city-wide quintiles) or 5-category variables (for proximity to parks and transit stops). Second, all covariates reported by other researchers as being correlated with objective and perceived measures of the built environment (age, sex, education level, SES, marital status, and meeting PA recommendations) (4,6,10,27) were introduced into the models. Because of high levels of crime in Cuernavaca (28), models were also adjusted for perceived safety from crime in the neighborhood (perceived park safety for the model of proximity to parks) to control for potential confounding. To test if any feature modified the relationship between perceived and objective measures, we tested for interactions between objective measures and individual variables (including perceived safety) in the adjusted models. Models were run assuming robust standard errors, tested for specification error by using the Stata *linktest* procedure, and tested for multicollinearity by exploring the variance-inflation factor. Adjusted predictions and 95% CIs evaluated at the mean of the covariates were calculated using the post estimation command *margins. *Plots of predicted values were generated with these data by using the *marginsplot* post estimation command. All analyses accounted for the complex multistage clustered design and were weighted for probability of selection. Analyses were carried out using Stata v.13.0 (StataCorp LP) survey procedures.

## Results

No significant differences in sociodemographic features were found between participants originally recruited and the analytic samples. Participants were aged 42 years (95% CI, 40.7–43.2 y) on average. Of the 645 participants, 51.4% were female, 65.6% were married or cohabiting, 54.8% owned a motor vehicle, and 58.7% met the international recommendations of 150 minutes per week of moderate to vigorous PA (Table 1). Almost 95% perceived a transit stop within 10 minutes or less of walking distance, 49.1% perceived moderate intersection density, and more than 58.0% perceived having 10 or more destinations within a 10-minute walk (Table 2).

**Table 1:** Sociodemographic Characteristics^a^ of Mexican Adults (N = 645) Participating in Study on Features of Their Neighborhood Environment, Cuernavaca, Mexico, 2011

Variable	No. of Participants	% (95% Confidence Interval)^b^
**Sex**		
Female	353	51.4 (44.1–58.6)
Male	292	48.6 (41.4–55.9)
**Age, y**		
<35	210	33.0 (29.1–37.1)
35–50	250	38.8 (35.5–42.3)
>50	185	28.1 (24.1–32.7)
**Socioeconomic status^c^**		
Low	192	31.1 (23.7–39.6)
Medium	156	23.8 (20.3–27.6)
Medium-high	189	29.1 (24.3–34.4)
High	108	16.0 (12.8–19.8)
**Education**		
Elementary school or less	101	15.3 (12.4–18.9)
Some or complete middle school	159	25.5 (21.6–29.8)
Some or complete high school	177	26.8 (23.3–30.6)
Some or complete college	167	26.9 (23.3–30.8)
Post-graduate	41	5.5 (3.6–8.3)
**Motor vehicle ownership**		
No	290	45.2 (38.4–52.1)
Yes	355	54.8 (47.4–61.0)
**Marital status**		
Single	153	24.4 (21.3–27.8)
Married or cohabitating	421	65.6 (61.5–69.6)
Separated or divorced	54	7.5 (5.7–9.8)
Widowed	17	2.5 (1.4–4.4)
**Meet physical activity recommendations^d^**		
No	278	41.3 (36.7–46.1)
Yes	367	58.7 (53.9–63.3)

a All data based on self-report except data on meeting physical activity recommendations.

b Weighted for survey design. Percentages do not correspond exactly to frequencies.

c Categories based on 25 questions on household features and assets used by the National Health and Nutrition Surveys of Mexico (23).

d Minutes per week of moderate to vigorous physical activity were measured by accelerometers using 60-second epochs and were scored using the cut points for adults defined by Freedson et al (24).

**Table 2:** Objective and Perceived Measures^a^ of Selected Features of Neighborhood Environments Among Mexican Adult Survey Participants (N = 645), Cuernavaca, Mexico, 2011

Feature	Objective Measure			Perceived Measure		
	Variable	No. of Respondents	% (95% CI)^b^	Variable	No. of Respondents	% (95% CI)^b^
Residential density	**No. of residential units^c^**			**ANEWS residential density score^d^**		
	<276	130	19.1 (11.6 to 29.8)	<14	156	24.8 (20.0 to 30.4)
	277 to 396	127	21.5 (15.8 to 28.6)	15 to 25	147	23.3 (18.0 to 29.5)
	397 to 543	130	24.6 (17.8 to 33.0)	26 to 39	99	16.0 (13.1 to 19.4)
	544 to 765	127	18.3 (11.1 to 28.7)	40 to 74	130	21.0 (15.8 to 27.5)
	>765	131	16.4 (8.9 to 28.2)	≥75	113	14.9 (10.0 to 21.7)
Intersection density	**No. of ≥3-way street intersections^c^**			**Intersection density score^e^**		
	<107	128	20.5 (13.1 to 30.5)	1	48	7.4 (5.1 to 10.8)
	107 to <144	130	20.5 (13.8 to 29.3)	1.1 to 1.5	78	11.9 (8.9 to 15.8)
	144 to <187	130	22.4 (16.1 to 29.3)	1.6 to 2.3	324	49.1 (43.5 to 54.7)
	187 to <244	128	18.4 (11.2 to 28.8)	2.4 to 3.1	185	29.9 (25.1 to 35.2)
	≥244	129	18.2 (10.4 to 30.0)	3.2 to 4	10	1.6 (0.9 to 3.0)
Land-use mix	**Entropy score^f^**			**No. of destinations within a 10-min walk**		
	<−36	126	20.5 (11.1 to 34.9)	<7	126	19.8 (14.1 to 27.2)
	−36 to <−12	129	15.5 (8.8 to 25.9)	7 to 9	138	22.2 (18.6 to 26.2)
	−12 to <2.6	129	20.5 (13.5 to 29.9)	10 to 12	137	21.6 (17.5 to 26.4)
	2.6 to <15	131	21.1 (13.6 to 31.1)	13 to 15	139	22.6 (17.3 to 29.0)
	≥15	130	22.4 (12.3 to 37.3)	≥16	105	13.8 (9.8 to 19.0)
Proximity to parks	**Walking time to the nearest park, min**			**Walking time to the nearest park, min**		
	<5	211	22.8 (13.9 to 35.1)	<5	166	27.3 (19.0 to 37.6)
	6 to 10	141	25 (16.4 to 36.1)	6 to 10	86	13.8 (10.0 to 18.7)
	11 to 20	164	32.5(20.1 to 47.9)	11 to 20	138	21.1 (16.8 to 26.2)
	21 to 30	58	10.7 (5.1 to 20.9)	21 to 30	119	19.0 (13.9 to 25.4)
	>30	71	9.1 (3.3 to 22.5)	>30	136	18.8 (12.8 to 26.9)
Proximity to transit stops	**Walking time to the nearest transit stop, min**			**Walking time to the nearest transit stop, min**		
	<5	435	65.1 (50.1 to 77.6)	<5	522	81.2 (75.5 to 85.9)
	6 to 10	109	19.2 (12.0 to 29.3)	6 to 10	90	13.7 (10.1 to 18.1)
	11 to 20	51	8.7 (3.8 to 18.7)	11 to 20	28	4.7 (3.0 to 7.3)
	21 to 30	37	4.8 (0.2 to 12.3)	21 to 30	1	0.0 (0.0 to 0.0)
	>30	21	2.3 (0.0 to 10.6)	>30	4	0.0 (0.0 to 0.1)

Abbreviations: ANEWS, Abbreviated Neighborhood Environment Walkability Scale; CI, confidence interval.

a See Appendix for detailed definitions of all variables.

b Weighted for probability of selection. Percentages do not correspond exactly to frequencies.

c Measure estimated within a 500-m buffer surrounding participant’s home.

d Theoretical range 1–1,000; higher values indicate higher residential density.

e Theoretical range 1–4; higher values indicate higher intersection density.

f Higher entropy values indicate higher level of mixed-land use.

We found significant correlations between perceived and objective measures of residential density, land-use mix, proximity to parks, and proximity to transit stops (*P* < .001 for all correlations); perceptions of intersection density were not significantly correlated with objective measures (Table 3).

**Table 3:** Correlations Between Objective Measures of Selected Features of Neighborhood Environments and Perceptions About Those Features Among Mexican Adult Survey Participants (N = 645), Cuernavaca, Mexico, 2011

Built environment feature	Variable^a^	Mean^b^ (Range)	ρ^c^	*P* Value
**Residential density**				
Objective	Number of residential units within the 500-m buffer^d^	517.7 (68.6 to 1,906.0)	0.26	<.001
Perceived	ANEWS residential density score^d^	40.8 (33.1 to 48.5)		
**Intersection density**				
Objective	Intersection density (3-way or more) within the 500-m buffer	170.9 (12.1 to 393.4)	0.01	.80
Perceived	ANEWS street connectivity score	2.1 (1 to 4)		
**Land-use mix**				
Objective	Entropy score within the 1-km buffer	1.35 (−63.4 to 67.4)	0.22	<.001
Perceived	Number of destinations within 10-min walk	10 (0 to 23)		
**Proximity to parks**				
Objective	Walking distance to the nearest park, min	12.8 (0.0 to 41.4)	0.19	<.001
Perceived	Walking distance to the nearest park, min	18.1 (2.5 to 35)		
**Proximity to transit stops**				
Objective	Walking distance to the nearest transit stop, min^d^	6.0 (0.01 to 34.3)	0.16	<.001
Perceived	Walking distance to the nearest transit stop, min^d^	3.9 (2.5 to 35)		

Abbreviations: ANEWS, Abbreviated Neighborhood Environment Walkability Scale.

a See Appendix for detailed definitions of all variables.

b Weighted for survey design.

c Determined by using Pearson correlations weighted for survey design.

d Variables were log-transformed before running Pearson correlations.

**Figure 1:**
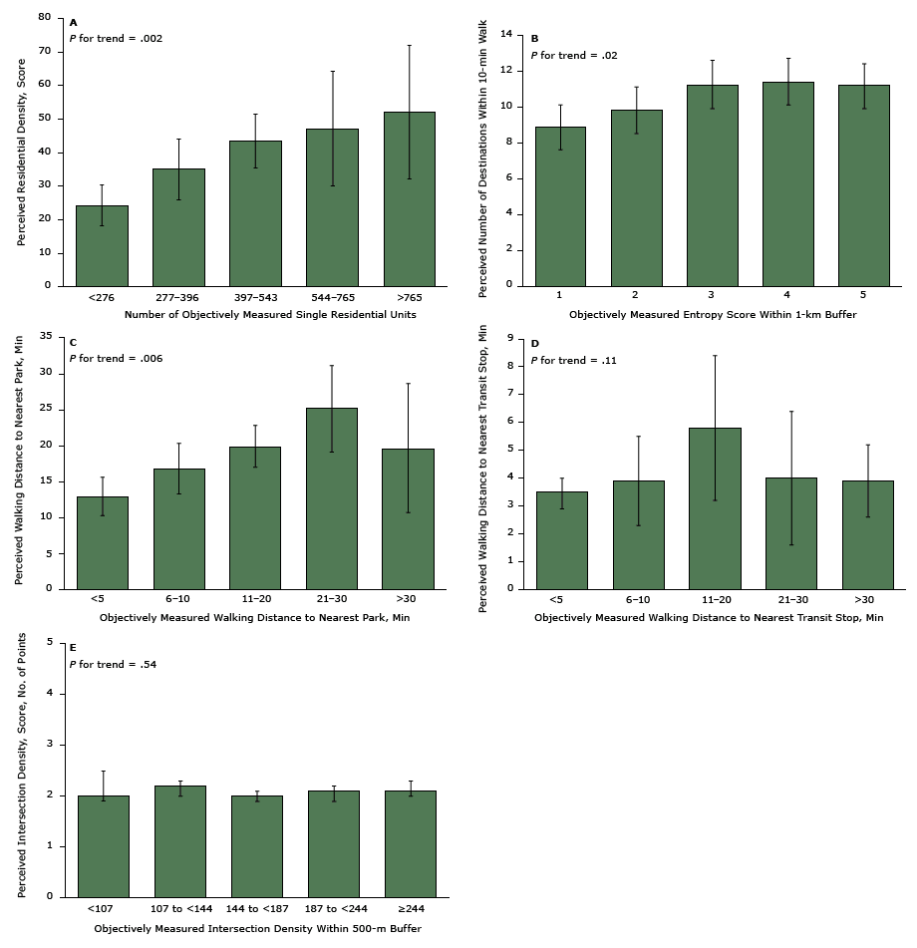
Associations between objectively measured and perceived measures of environmental features: A. Residential density, as determined by the number of single residential units (objectively measured) and a residential density score (perceived), calculated according to the protocol of the Abbreviated Neighborhood Environment Walkability Scale (theoretical range, 0–1,000); B. Land-use–mix, as determined by an entropy score (objectively measured) and the number of destinations within a 10-minute walk (perceived); C. Walking distance to nearest park in minutes, objectively measured and perceived (theoretical range, 2.5–35 min); D. Walking distance to nearest transit stop in minutes, objectively measured and perceived (theoretical range, 2.5–35 min); and E. Intersection density as determined by objective measurement and a score of perception (theoretical range, 1–5, based on averaged scores for Likert-scale response options of 1, strongly disagree, to 5, strongly agree to 2 statements: “There are many alternative routes for getting from place to place in my neighborhood” and “The distance between intersections in my neighborhood is usually short.”). Details of measurements are provided in the Appendix. Adjusted predictions and 95% confidence intervals (CIs) were estimated after running adjusted regression models. Models were adjusted for sex, age, socioeconomic status, motor-vehicle ownership, education level, perceived safety in the neighborhood, years living in the neighborhood, and corresponding interaction terms for each calculation. Error bars are 95% CIs. **Table:** 

Unadjusted models for estimating the association between perceived and objectively measured variables showed significant relationships between categories or quintiles of objectively measured residential density, land-use mix, proximity to transit stops, and proximity to parks and their corresponding perceived variable (*P* value for trend across categories < .05). After adjusting for covariates, the magnitude, direction, and significance of the relationships did not change for residential density (Figure 1A), land-use mix (Figure 1B), or proximity to parks (Figure 1C). The adjusted models showed that higher quintiles of the objective variable were associated with increases in the corresponding perceived variable (*P* value for trend across categories < .05). However, we found no differences in perceived number of destinations or walking distance to the nearest park among the three highest categories of the corresponding objective variable (*P* value > .05 between each category) (Figure 1B and Figure 1C). Adjusted associations showed that the nearest parks (within a 10-min walk per the objective measure) were perceived as being farther away than they actually were, whereas the opposite was true for the farthest parks (≥30-min walk per the objective measure). The adjusted model of proximity to transit stops showed that perceived walking distances were similar for the nearest transit stops (within 5-min walk and 5–10 min walk per the objective measure) and farthest transit stops (21–30-min and >30-min walking per the objective measure); participants perceived transit stops physically located at medium distance (11–20-min walk per the objective measure) as farther away than those at the closest distance (within 5 min walk per the objective measure) (Figure 1D). No significant adjusted relationships between objective and perceived measures of intersection density were observed (Figure 1E).

Objectively Measured	Perceived
**Figure 1A**	
No. of single residential units	Residential density score (95% CI)
<276	24.2 (18.1–30.4)
277–396	35.1 (26.1–44.1)
397–543	43.5 (35.5–51.4)
544–765	47.1 (30.0–64.2)
>765	52.1 (32.2–72.1)
**Figure 1B**	
Entropy score within 1-km buffer	No. of destinations within 10-min walk (95% CI)
1	8.9 (7.6–10.1)
2	9.8 (8.5–11.1)
3	11.2 (9.9–12.6)
4	11.4 (10.1–12.7)
5	11.2 (9.9–12.4)
**Figure 1C**	
Walking distance to the nearest park, min	Walking distance to the nearest park, min (95% CI)
<5	12.9 (10.3–15.6)
6–10	16.8(13.3–20.3)
11–20	19.8 (17.0–22.8)
21–30	25.3 (19.1–31.1)
≥30	19.6 (10.7–28.6)
**Figure 1D**	
Walking distance to the nearest transit stop, min	Walking distance to the nearest transit stop, min (95% CI)
<5	3.5 (2.9–4.0)
6–10	3.9 (2.3–5.5)
11–20	5.8 (3.2–8.4)
21–30	4.0 (1.6–6.4)
≥30	3.9 (2.6–5.2)
**Figure 1E**	
Intersection density within 500-m buffer	Intersection density score (95% CI)
<107	2.0 (1.9–2.5)
107 to <144	2.2 (2.0–2.3)
144 to <187	2.0 (1.9–2.1)
187 to <244	2.1 (1.9–2.2)
≥244	2.1 (2.0–2.3)

### Individual features and perception of the built environment

**Figure 2:**
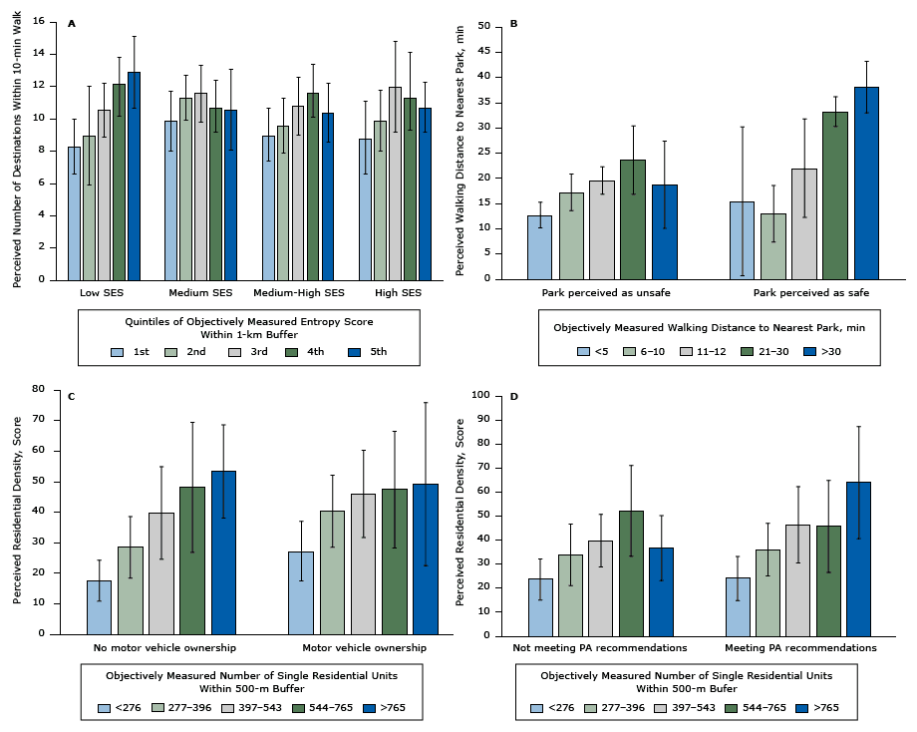
Individual features and perceptions of the built environment. Adjusted predictions and 95% CIs estimated after running adjusted regression models. Models were adjusted for sex, age, SES, motor-vehicle ownership, education level, perceived safety in the neighborhood, years living in the neighborhood, as well as the corresponding interaction terms for each figure. Error bars are 95% confidence intervals (CIs). Abbreviations: SES, socioeconomic status; PA, physical activity. **Table:** 

Individual variables significantly modified the relationship between objective and perceived measures of the built environment (Figure 2). Low-SES participants reported more destinations within less than a 10-minute walk as quintiles of objectively measured land-use mix increased (*P* for trend across categories < .05); this trend was not observed for other levels of SES (Figure 2A). Although participants perceiving parks as safe reported longer walking distances as quintiles of objectively measured walking distances increased (*P* for trend across categories = .02), participants perceiving parks as unsafe reported significantly shorter walking distances for parks located 21–30 minutes or more than 30 minutes away (*P* < .05) (Figure 2B). Compared with participants who did not own a motor vehicle, participants who owned a motor vehicle reported higher residential density scores for the first 4 quintiles of objectively measured residential units (Figure 2C). Participants meeting PA recommendations reported higher residential density scores as quintiles of objectively measured residential density increased (*P* for trend across categories < .01); this relationship was not observed for those not meeting PA recommendations (Figure 2D). No other individual features modified the relationships between objective and perceived measures of the built environment.

Figure	Strata	
	Adjusted Prediction (95% CI)	Adjusted Prediction (95% CI)
**Figure 2A**	**Perceived number of destinations within 10-min walk**	
Quintiles of objectively measured entropy score within 1-km buffer	**Low SES**	**Medium SES**
1	8.3 (6.6–10.0)	9.9 (8.0–11.7)
2	9.0 (5.9–12.0)	11.3 (9.9–12.7)
3	10.6 (8.9–12.2)	11.6 (9.8–13.3)
4	12.2 (10.2–14.1)	10.7 (9.2–12.1)
5	12.9 (10.7–15.1)	10.6 (8.0–13.1)
Quintiles of objectively measured entropy score within 1-km buffer	**Medium-High SES**	**High SES**
1	9.0 (7.4–10.7)	8.8 (6.6–11.1)
2	9.6 (7.9–11.3)	9.9 (8.0–11.8)
3	10.8 (9.0–12.6)	12.0 (9.2–14.8)
4	11.6 (10.1–13.1)	11.3 (9.3–13.4)
5	10.4 (8.6–12.2)	10.7 (9.2–12.3)
**Figure 2B**	**Perceived walking distance to the nearest park (min)**	
Objectively measured walking distance to the nearest park, min	**Park perceived as unsafe**	**Park perceived as safe**
<5	12.7 (10.1–15.2)	15.4 (0.6–30.1)
6–10	17.2 (13.5–20.8)	13.0 (7.4–18.6)
11–20	19.6 (16.9–22.3)	22.0 (12.2–31.7)
21–30	23.7 (17.0–30.5)	33.2 (30.2–36.1)
≥30	18.7 (10.0–27.3)	38.1 (33.0–43.2)
**Figure 2C**	**Perceived residential density score**	
Objectively measured number of single residential units within 500-m buffer	**No motor vehicle ownership**	**Motor vehicle ownership**
<276	17.6 (10.9–24.3)	27.3 (17.5–37.0)
277–396	28.5 (18.4–38.6)	40.3 (28.5–52.1)
397–543	39.8 (24.6–54.9)	46.0 (31.7–60.3)
544–765	48.2 (26.9–69.4)	47.4 (28.3–66.5)
>765	53.4 (38.1–68.6)	49.2 (22.5–75.9)
**Figure 2D**	**Perceived residential density score**	
Objectively measured number of single residential units within 500-m buffer	**Not meeting PA recommendations**	**Meeting PA recommendations**
<276	23.7 (15.1–32.2)	24.1 (14.9–33.2)
277–396	33.9 (21.1–46.7)	36.1 (25.1–47.0)
397–543	39.8 (28.9–50.8)	46.4 (30.5–62.3)
544–765	52.2 (33.3–71.1)	45.7 (26.6–64.9)
>765	36.7 (23.2–50.2)	64.0 (40.6–87.3)

## Discussion

We found weak correlations between objective and perceived measures of residential density, land-use mix, proximity to parks, and proximity to transit stops; perceived and objective measures of intersection density were not correlated. Adjusted associations suggest that perceived land-use mix and proximity to parks may reach a high stable level, regardless of additional increases in the objective variable, and that perceived proximity to transit stops and intersection density were not explained by our objective measure. Our results also indicate that the relationship between objective and perceived data are neither linear nor uniform across individual or neighborhood factors. The low level of concordance between objective and perceived measures of built environment features confirm that perceptions should not be considered as proxies for objective measures.

In settings with many and varied environmental features, perceived measures may not reflect the variability of objective measures. According to a study that examined variation in the built environment by using geographic information systems in 12 countries and 15 cities, Cuernavaca has the second-highest intersection density of 15 cities (29). Cuernavaca also has a high density of transit stops (29) Although the public transportation system in Cuernavaca has official transit stops, buses stop whenever and wherever a rider signals to the driver. In this study, the difference in mean perceived walking distance between the first and fifth quintile of the corresponding objective measure was only one minute. Future studies conducted in settings where environmental features are uniformly dense are needed to confirm whether the variability of objectively measured features corresponds with participants’ perceptions.

Previous reports identified groups of people in which stronger associations between perceived and objectively measured data were found (4,6,10). Inconsistencies among individual factors have been explained by the degree of environmental exposure among individuals. People who interact more with their neighborhood are hypothesized to better understand their neighborhood’s characteristics. For example, active people may spend time walking in their neighborhood, which would give them a familiarity with their environment that less active people do not have (6,10). Higher levels of exposure allow people to acquire a better understanding of their surroundings and therefore provide more accurate reports. This idea is in line with our findings, which suggest that participants who meet PA recommendations and participants who did not own a motor vehicle had a better awareness of their neighborhood.

Contrary to findings of studies conducted in high-income countries, which indicate that correspondence between objective and perceived environmental features is lower among people with low SES compared with people who have higher SES (6), we found that participants who had low SES had a better awareness of neighborhood destinations than did participants who had higher SES levels. According to our results, low-SES participants reported more destinations within a 10-minute walk as quintiles of objectively measured land-use mix increased, but this trend was not observed for higher levels of SES. One possible explanation is that motivation for PA may differ between people in Mexico and people in high-income countries. Data from the IPEN (International Physical Activity and the Environment Network) adult study suggest that PA in Mexico is more strongly driven by necessity (transportation) than by choice (leisure) (15). Low-income people may be active by necessity, especially for transportation. Data on transportation for our sample suggest that low-income participants in Cuernavaca engage in approximately 100 minutes more of transportation activity than do high-income participants (A.J., D.S., M.P., unpublished data, 2016). The increased exposure to their neighborhood environment caused by active transportation may explain a better awareness of destinations among low-income participants in our study.

Likewise, participants perceiving parks as safe provided better estimates on walking distance to parks. Perceptions of certain neighborhood features, such as neighborhood cohesion, are related to better correspondence between perceived and objectively measured distance to parks (8). People probably do not often visit a park that has unattractive features, such as poor perceived safety (30), and therefore they may provide poor estimates of proximity. Participants who visit parks may not perceive them as unsafe. To understand these relationships, studies are needed on the use, perceived safety, and perceived proximity of parks. Previous studies on environmental correlates of PA among Mexican adults showed that objectively measured distance to parks was not associated with PA when parks were perceived as safe but was negatively correlated when parks where perceived as unsafe (12). In contrast, a previous analysis of our sample showed that perceived proximity to parks was the strongest correlate for PA regardless of the perception of park safety (31). Taken together, these findings suggest that perceived proximity to parks is a more proximal correlate of PA and that the way in which proximity to parks is perceived may be moderated by perceived park safety. Path analysis may be useful for testing this hypothesis (32). Future research should examine the influence of park features, park use, and park-related PA to improve strategies to increase awareness and use of parks.

This study has several limitations. Available environmental measures were not entirely comparable. Our variable for perceived land-use mix considered 23 types of destinations, whereas the entropy score is a composite of only 3 destination types. Although we confirmed the accuracy of the shapefile provided by INEGI containing the counts and locations of city parks and other destinations, the entropy score was calculated by using a land-cover land-use map instead of a parcel-level land-use map, which may have increased the inaccuracy of our measure. Additionally, informal commerce (eg, street vendors, residential space used for commerce) is common in Mexican cities, and data on such commercial activity are not captured by the GIS-based measure to which we had access. Associations between the objective and perceived environment depend on the congruence between neighborhood definitions. We tried to account for neighborhood size by creating variables with similar walking distances to various destinations (eg, parks, transit stops, grocery stores). Nonetheless, we could not create these variables for intersection density or residential density. For these variables, objective measures were derived by using 500-m buffers. Studies in high-income countries show that similar buffers (approximately 400 m) adequately capture data on perceptions among adults of neighborhood walkability (9,18,19). However, no evidence exists on the optimal buffer size in Latin America. Therefore, we cannot determine whether weak associations are due to a mismatch between definitions of neighborhood size or to genuine misperceptions about the neighborhood. When we tested correlations and associations using objective measures derived by using 1-km buffers, the results were similar. 

This study also has strengths. It is the first study to examine the relationship between perceived and objective measures of the built environment in a middle-income country. Other strengths are our representative population; our use of cross-validated, comparable measures of perceived environmental features; and our use of objective GIS data.

We found weak correlations in Cuernavaca between perceived and objective measures of 5 environmental features related to PA in high-income countries. Our study confirms results from studies in high-income countries indicating that associations between perceived and objective measures are modified by individual sociodemographic and psychosocial factors, such as perception of safety. It provides guidance for researchers wanting to explore the environmental correlates of physical activity, suggesting that perceived measures of residential density, land-use mix, and proximity to parks may be used. However, when studying a city like Cuernavaca, researchers should not assume that perceived measures of intersection density and proximity to transit stops are the same as objective measures. In an environment in which levels of intersection density and transit-stop density are uniformly high, these variables may not be useful for understanding variability in PA.

Our results highlight the relevance of contextual factors when studying PA. Although some variables derived from research in high-income countries may be useful in understanding the environmental determinants for PA in Cuernavaca, a new set of variables consistent with the environment and culture in Mexico could better predict variability in PA. Continued research can identify such variables.

Finally, our findings also suggest that policies aimed at increasing the availability and access of neighborhood features for PA may not be sufficient to increase PA among residents (11) Complementary activities to improve perceptions of the environment should be undertaken, particularly targeted toward groups of people whose perceptions of environmental features are in least agreement with objectively measured features.

## Appendix Operationalization of Perceived and Objective Measures of Features of the Built Environment

**Table:**

Built environment feature	Perceived variable	Objective variable
Residential density	ANEWS asks participants to report how common were 6 types of residential buildings (from single-family residences to ≥13-story buildings) in their neighborhood. Five response options were provided, from none, coded as zero, to all, coded as 4. We calculated a residential density score using the following formula, as per the ANEWS protocol (21): single-family detached + (12 × row houses or townhouses with 1–3 stories) + (10 × apartments or condominiums with 1–3 stories) + (25 × apartments or condominiums with 4–6 stories) + (50 × apartments or condominiums with 7–12 stories) + (75 × apartments or condominiums with ≥13 stories).	We calculated the number of residential units within the 500-m buffer.
Land-use–mix	ANEWS asked participants to report time walking from home to 23 different types of nonresidential destinations: 1) convenience/small grocery store, 2) supermarket, 3) hardware store, 4) fruit/vegetable market, 5) laundry/dry cleaners, 6) clothing store, 7) post office, 8) library, 9) elementary school, 10) other schools, 11) book store, 12) fast food restaurant, 13) coffee place, 14) bank/credit union, 15) non-fast food restaurant, 16) video store, 17) pharmacy/drug store, 18) salon/barber shop, 19) participant’s job or school, 20) bus or trolley stop, 21) park, 22) plaza, 23) gym or fitness facility. Response options for these items were scored according to a Likert scale as follows: 1 (1–5 min), 2 (6–10 min), 3 (11–20 min), 4 (21–30 min), and 5 (≥31 min). We calculated the number of reported destinations within a 10-minute walk (corresponding to walking approximately 1 km at 5 km/h) (22).	We calculated land-use–mix diversity by generating an entropy score within the 1-km buffer with the following formulae: −1 × {[∑(*pi*)(ln *pi*)]/ln *k*}, where *p* = proportion of total land uses, *i* = land use category, ln = natural logarithm, and *k* = number or land uses (14). Higher scores indicate higher land-use diversity.
Intersection density	ANEWS items included the following: 1) the distance between intersections in my neighborhood is usually short, 2) there are many alternative routes for getting from place to place in my neighborhood. Five response options were available, from strongly disagree, coded as 1, to strongly agree, coded as 5. These response options were recoded so that higher values indicated higher levels of intersection density. We computed the intersection density score by averaging the scores reported on the 2 items.	We estimated the number of 3-way or more street intersections per buffer area within the 500-m buffer.
Proximity to parks	We used the individual item referring to parks from the list of 23 nonresidential destinations of the ANEWS land-use–mix section. Participants reported the walking distance to the nearest park as follows: 1 (1–5 min), 2 (6–10 min), 3 (11–20 min), 4 (21–30 min), and 5 (≥31 min). We replaced response options 1, 2, 3, 4, and 5 by 2.5, 7.5 min, 15 min, 25 min, and 35 min, respectively.	We estimated the distance from the participant’s home to the nearest park by using the street network. We used this information to calculate the walking time to the nearest park assuming a walking speed of 5 km/h (22). We categorized participants as follows: 1 (1–5 min), 2 (6–10 min), 3 (11–20 min), 4 (21–30 min), 5 (≥31 min).
Proximity to transit stops	We used a similar approach to the one used for perceived proximity to parks to estimate the perceived proximity to the nearest transit stop. Participant responses to the individual item referring to transit stops were operationalized in the same way as reported walking distance to the nearest park.	Distance from the participant’s home to the nearest street-corner intersecting a bus route by using the street network. We calculated categories and walking time to the nearest transit stop using the same methodology that we used for proximity to parks.
